# Identification of a novel metabolic engineering target for carotenoid production in *Saccharomyces cerevisiae* via ethanol-induced adaptive laboratory evolution

**DOI:** 10.1186/s40643-021-00402-5

**Published:** 2021-06-11

**Authors:** Buli Su, Anzhang Li, Ming-Rong Deng, Honghui Zhu

**Affiliations:** Guangdong Microbial Culture Collection Center (GDMCC), Guangdong Provincial Key Laboratory of Microbial Culture Collection and Application, State Key Laboratory of Applied Microbiology Southern China, Institute of Microbiology, Guangdong Academy of Sciences, Guangzhou, 510070 People’s Republic of China

**Keywords:** *Saccharomyces cerevisiae*, Adaptive laboratory evolution, Carotenoid, *PFK1*, Reverse engineering, Cell wall

## Abstract

Carotenoids are a large family of health-beneficial compounds that have been widely used in the food and nutraceutical industries. There have been extensive studies to engineer *Saccharomyces cerevisiae* for the production of carotenoids, which already gained high level. However, it was difficult to discover new targets that were relevant to the accumulation of carotenoids. Herein, a new, ethanol-induced adaptive laboratory evolution was applied to boost carotenoid accumulation in a carotenoid producer BL03-D-4, subsequently, an evolved strain M3 was obtained with a 5.1-fold increase in carotenoid yield. Through whole-genome resequencing and reverse engineering, loss-of-function mutation of phosphofructokinase 1 (*PFK1*) was revealed as the major cause of increased carotenoid yield. Transcriptome analysis was conducted to reveal the potential mechanisms for improved yield, and strengthening of gluconeogenesis and downregulation of cell wall-related genes were observed in M3. This study provided a classic case where the appropriate selective pressure could be employed to improve carotenoid yield using adaptive evolution and elucidated the causal mutation of evolved strain.

**Supplementary Information:**

The online version contains supplementary material available at 10.1186/s40643-021-00402-5.

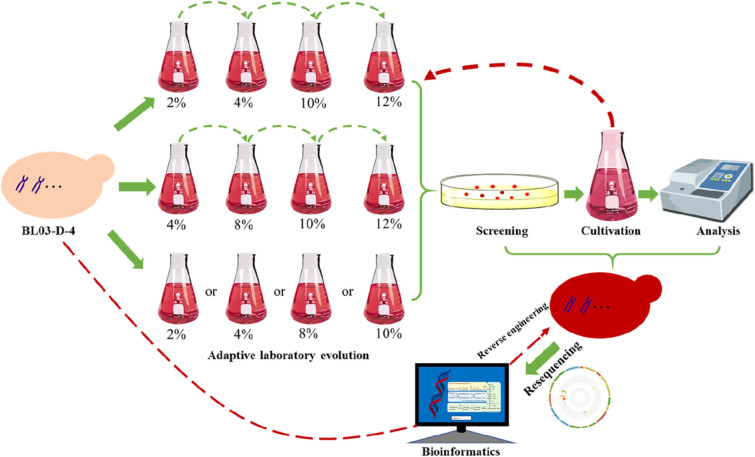

## Introduction

Carotenoids are a large kind of isoprenoid pigment that can be used in many fields such as colorants, antioxidants, and nutrients (Saini and Keum 2019). Currently, carotenoids are produced by chemical synthesis or extraction from natural sources, which are mostly restricted by complicated structures and shortage of raw materials (Mussagy et al. 2019). Wild-type *Saccharomyces cerevisiae* does not normally accumulate carotenoids but engineered strains, obtained through rational engineering, have achieved enhanced carotenoid yield in *S. cerevisiae* (Wang et al. 2019). Many strategies have been applied and high levels of yields were obtained (Chen et al. 2016; Hong et al. 2019; Shi et al. 2019; Xie et al. 2015), while the highest yield of 73 mg/g CDW was achieved through lipid engineering (Ma et al. 2019). However, it was difficult to discover new targets that were relevant to the heterologous production of carotenoids and, furthermore promote the production of carotenoids in recombinant *S. cerevisiae* based on previous studies. Fortunately, random disturbance in the genes at a whole-genome scale (through mutagenesis or evolution) provided a choice for acquiring a carotenoid hyper-producer (Zhu et al. 2018). For instance, using a single atmospheric and room temperature plasma (ARTP) treatment, astaxanthin yield was improved by 0.83-fold over the parental strain and three potential targets related to astaxanthin biosynthesis in yeast were revealed (Jin et al. 2018). Furthermore, a combined strategy composing physical mutagenesis by ARTP and adaptive laboratory evolution (ALE), driven by hydrogen peroxide, was executed and the titer of astaxanthin was increased nearly fourfold (Jiang et al. 2020).

ALE is a widely used technology to achieve insights into the mechanisms of adaptive mutations that accumulate under designed culture conditions for long periods of screening (Dragosits and Mattanovich 2013). ALE has been proven to be a potent tool in metabolic engineering, both for the revelation of new design principles and the engineering of excellent strains (Zhu et al. 2018). Many superior phenotypes have been achieved through ALE, such as improvement of the growth rate and production, the adaption of strains to utilize non-native substrates, and enhancement of the resistance towards environmental stresses (Sandberg et al. 2019). Furthermore, the relationship between genomic changes and the excellent phenotype could be uncovered by whole-genome resequencing combined with systems biology (Yang et al. 2019). For example, ALE has been applied to adapting hydrogen peroxide-tolerant yeast to improve carotenoids production (Reyes et al. 2014), and the beneficial mutations led to increased yield have been revealed (Godara et al. 2019). Compared to random mutagenesis approaches such as ARTP and ultraviolet light, ALE with sequential passages is a reasonably easy technique for confirming crucial mutations related to the beneficial phenotype due to its low mutation probability. Furthermore, the production of the target product could be improved by introducing the identified mutations into an engineered producer (Lee and Kim 2020).

ALE used suitable stress as a driving force for screening mutants with superior phenotypes. Because carotenoid yield was facilitated by engineering microbial membranes which could also increase ethanol tolerance (Guo et al. 2020; Hong et al. 2019), we hypothesized that ethanol stress might be used as a driving force for the adaptation of yeast for increased carotenoids accumulation. As a proof of concept, ethanol-induced ALE was applied to a recently constructed *S. cerevisiae* strain, BL03-D-04, harboring the carotenoid synthetic pathway (Su et al. 2020b). Improvement of carotenoid yield was obtained through this novel ALE strategy to screen for higher carotenoid producers. The underlying cause related to the promotion of carotenoids accumulation in the evolved strain were assessed using whole-genome resequencing and transcriptome analysis. The inactivation of phosphofructokinase 1 (*PFK1*) was determined as the causal mutation of the evolved strain with improved carotenoid yield, and strengthening of gluconeogenesis and downregulation of cell wall-related genes were identified as the likely regulation that resulted in increased carotenoid yield.

## Material and methods

### Microorganisms and growth conditions

*S. cerevisiae* strain BL03-D-4, derived from BY4742 (*MATα, his3Δ1, leu2Δ0, lys2Δ0, ura3Δ0*), was chosen as the parental strain. All *S. cerevisiae* strains used in this study are listed in Additional file 1: Table S1. Primers are provided in Additional file 1: Table S2. For cultivation, a single colony was picked up from a fresh YPD plate and transferred to a 5 mL YPD medium. After being cultured at 200 rpm and 30 °C, 1 mL seed culture was inoculated into a 250 mL shake-flask with 50 mL YPD medium, or modified YPD medium (YPM) to an optical density (OD_600_) of about 0.05 and then incubated at 30 °C for 24 to 96 h. After incubation, the cultures were analyzed for biomass and carotenoid content. The YPD medium contained 20 g L^−1^ tryptone, 20 g L^−1^ glucose, and 10 g L^−1^ yeast extract (Oxoid, LOTs of 2,665,431–02). YPM medium contained 20 g L^−1^ tryptone, 20 g L^−1^ glucose, 10 g L^−1^ yeast extract (Angel, FM802, LOT: 2018082210C9), salt (10 g L^−1^ KH_2_PO_4_, 2.5 g L^−1^ MgSO_4_, 3.5 g L^−1^ K_2_SO_4_, 0.25 g L^−1^ Na_2_SO_4_) and 1 mL trace metal solution (TMS) described in previous literature (Su et al. 2020b).

### Adaptive laboratory evolution experiments

**Fig. 1:**
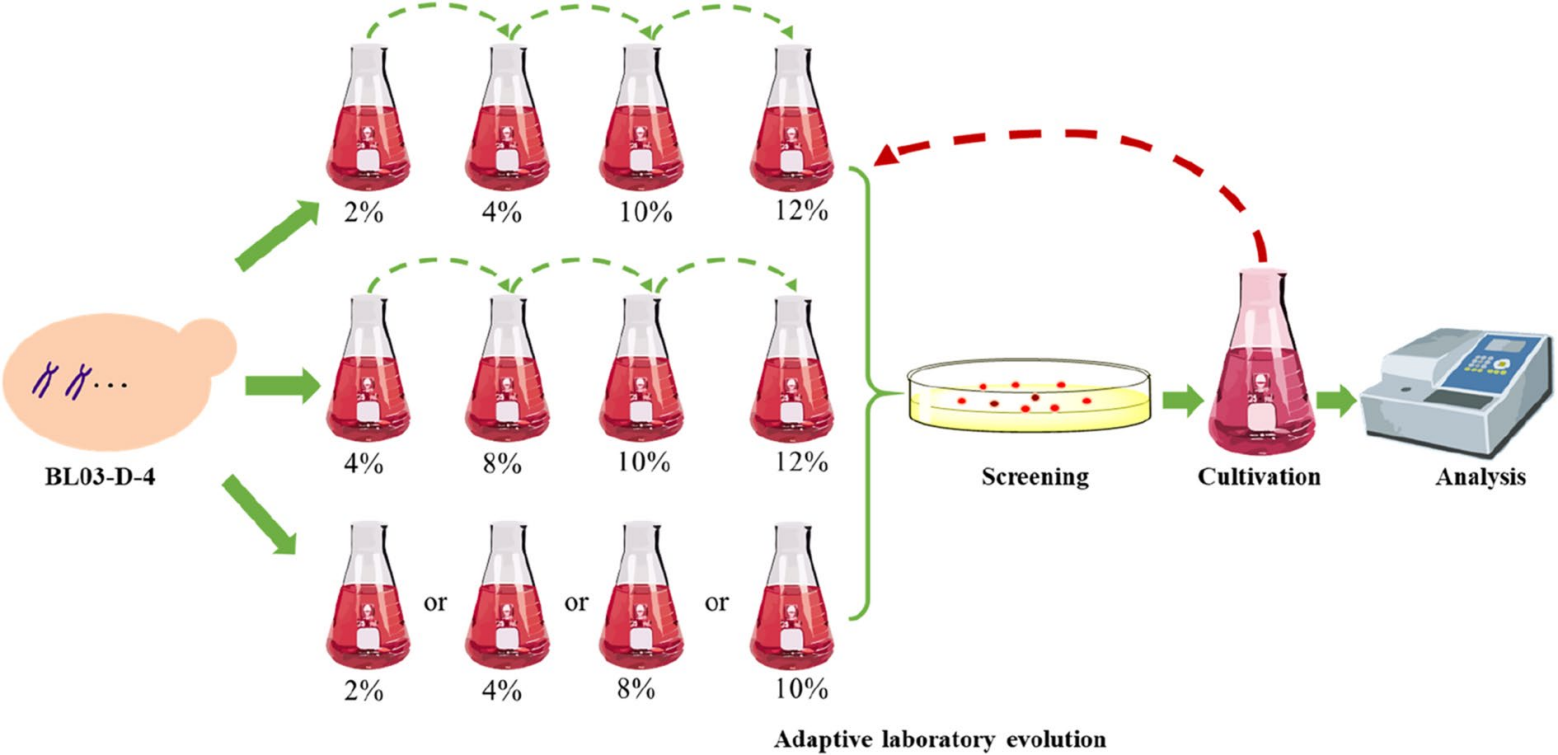
Schematic diagram of the ethanol-induced adaptive laboratory evolution. Gradually increasing (from 2 to 12% or from 4 to 12%) and specific concentrations (2%, 4%, 8%, and 10%) of ethanol were selected as the driving forces for the ALE. After screening and analytical certification, the excellent strain will be turned into another round of evolution. The mutations of excellent strain were uncovered through whole-genome resequencing and then parental strain was reverse engineered to gain a rationally designed strain

Sequential passages and batch cultures were performed using the parental strain (BL03-D-4) and YPD media with different concentrations of ethanol under the culture conditions illustrated in Fig. 1. Cells were inoculated into 50 mL YPD medium at OD_600_ of 0.05 to maintain the growth phase. After each cultivation, cells with appropriate concentrations were plated on YPD plates for single colony isolation, based on cell color. The single colonies obtained were inoculated in the YPD medium for further screening.

### Whole-genome resequencing and transcriptome analysis

The strains chosen for whole-genome resequencing were cultivated in 50 mL YPD medium at 30 °C in a shaker at 200 rpm for 24 h. Genomic DNA was extracted according to the manufacturer’s protocol using the HiPure Yeast DNA Kit (Magen, Guangzhou, China). At least 5 μg of each genomic DNA sample was provided to Shanghai Majorbio Bio-pharm Technology Co. Ltd, for sequencing using the Illumina HiSeq 2000 platform. Paired-end reads of ~ 250 bp were generated. The average sequencing depths of the samples were 70 to 90. Fastq DNA-seq raw data were deposited in the Genome Sequence Archive (GSA) server at the BIG Data Center (http://bigd.big.ac.cn, GSA accession No. CRA003704).

Total RNA from yeast cells was extracted according to our previous work (Su et al. 2020a). The concentrations and quality of the RNA samples were examined by Biospec-nano (Shimadzu, Kyoto, Japan). 1 μg total RNA sample was used for mRNA library preparation and RNA sequencing (Illumina HiSeq), performed by Shanghai Majorbio Bio-pharm Technology Co. Ltd. Fastq RNA-seq raw data were deposited into NCBI (GEO accession number GSE164470). Data processing was accomplished by the online Majorbio Cloud Platform (www.majorbio.com).

### Gene deletions

Auxotroph marker *HIS3* including a promoter and terminator, was amplified from the genomic DNA of *S. cerevisiae* S288C. The homologous arm (~ 50 bp) was designed in primers. For deletions of target genes, one-step integration of PCR-amplified deletion cassettes, including *HIS3*, was adopted (Chen et al. 2016). Primers PFK1-F and PFK1-R were used for amplification of *HIS3* from genomic DNA of *S. cerevisiae* S288C. Primers PFK1-F-2 and PFK1-R-2 were used for the addition of homologous arms targeted to *PFK1*. Primers PFK2-F and PFK2-R were used for amplification of *HIS3* from genomic DNA of *S. cerevisiae* S288C. Primers PFK2-F-2 and PFK2-R-2 were used for the addition of homologous arms targeted to PFK2. *PFK1* and *PFK2* deletions were verified by diagnostic PCR using paired primers PFK1-F-2/PFK1-CHECK-R and PFK2-F-2/PFK2-CHECK-R, respectively.

### Cell dry weight, specific growth rate and carotenoid quantification

Cell dry weight (CDW) was determined according to the OD_600_ value and correlated to CDW by the CDW/OD_600_ standard curve [y = 0.184x + 0.891 (x is OD_600_, y is CDW, R^2^ = 0.992]. The specific growth rate (μ) was calculated by the equation, μ = (ln X2 − ln X1)/(t2 − t1), where X1 and X2 are the biomasses at time t1 and t2, respectively.

For carotenoid quantification, strain culture after fermentation was transferred to a 1.5-mL sample tube and the cells were collected by centrifugation at 12,000 rpm for 5 min. The sedimentary cells were disrupted using 3 mol L^−1^ HCl and boiling for 4 min. Then, cell debris was washed thrice by sterile water, resuspended in acetone for extraction, and centrifuged at 12,000 rpm for 2 min. The supernatant was transferred to a new tube for quantification of total carotenoids using a UV/Vis spectrometer at 470 nm. The extinction coefficient was adopted using an A 1% 1 cm of 3450 (Su et al. 2020b).

### Morphological observation

The morphology of the strains was observed by a light microscope (DM6/MC190, Leica). In brief, the strains were cultivated in 50 mL YPD medium at 30 °C for 48 h, followed by centrifuging and washing with PBS three times. They were then observed through the microscope.

## Results

### Adaptive laboratory evolution of BL03-D-4 under ethanol stress

Because the heterologous accumulation of carotenoids in *S. cerevisiae* poses a metabolic burden on host cells, a suitable pressure was needed to endow the higher producers a striking feature (Reyes et al. 2014). Based on the already known mechanisms of ethanol tolerance (Ma and Liu 2010), we hypothesized that yeast strain would present different levels of carotenoids yield with the fluctuant composition of the membrane under ethanol stress, and there might be some status fitted to the carotenoids accumulation. Thus, in this study, an ethanol-induced ALE strategy was developed for improving carotenoid yield. For the ALE experiment, sequential and batch cultures were performed in YPD medium supplemented with different concentrations of ethanol (v/v) (Fig. 1). When the cell growth reached a plateau, the ALE process was terminated and the evolved cells were plated on YPD plates without ethanol to screen darkened colonies. Subsequently, an evolved strain M3 (deposited at Guangdong Microbial Culture Collection Center with GDMCC No. 61336) was obtained in the sequential cultures, through a range of adaptive experiments with gradually rising concentrations of ethanol (the first horizontal panel of Fig. 1 at 10% after three subcultures) based on darkened cell color (Additional file 1: Figure S1). Furthermore, M3 was also turned into another round of evolution. However, no better strain was obtained using the above ALE strategy.

**Fig. 2:**
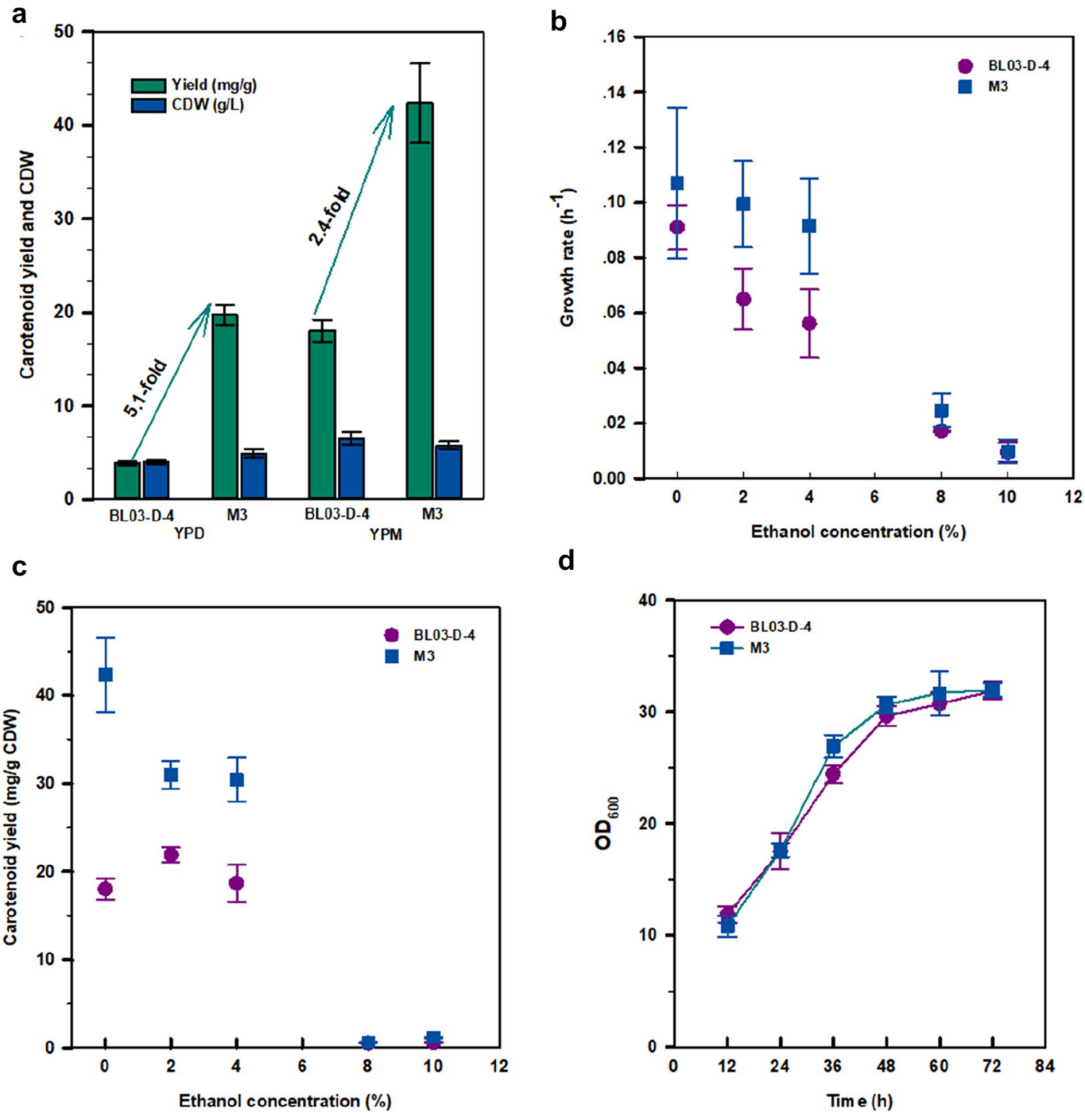
Characterization of parental (BL03-D-4) and evolved (M3) strains. Carotenoid yields and biomasses of BL03-D-4 and M3 in YPD and YPM media after 96 h of incubation (**a**). Specific growth rates of BL03-D-4 and M3 under different concentrations of ethanol (**b**). Effect of different concentrations of ethanol on carotenoid yields of BL03-D-4 and M3 (**c**). Growth curves of BL03-D-4 and M3 in YPM media without ethanol stress (**d**). Error bars represent standard deviations of three replicates

Shake-flask fermentation showed that the carotenoid yield of M3 increased about 5.1- and 2.4-fold compared with BL03-D-4 in YPD and YPM media, respectively (Fig. 2a). After 96 h of incubation, the carotenoid yield of M3 reached 42.4 mg/g CDW in the YPM medium (YPM medium facilitated carotenoid accumulation (Su et al. 2021)). The specific growth rate of strain M3 increased markedly, compared to that of the parental strain BL03-D-4 in the concentration from 2 to 8% ethanol (Fig. 2b). Both strains grew poor in the presence of 10% ethanol (Additional file 1: Figure S2). We also observed that the additional supplement of ethanol had a remarkably repressive effect on the carotenoid yield of M3 especially under a high concentration of ethanol (Fig. 2c). However, there was no significant difference in cell growth between BL03-D-4 and M3 during the cultivation without ethanol (Fig. 2d), indicating that the ALE process had no significant influence on the biomass of M3. Therefore, the improvement of carotenoids yield in M3 was not due to facilitating the strain growth, but was probably concerned with the change of metabolic pathway involved in genetic mutations and systematic transcriptional regulation, which needs to be further investigated.

### Whole-genome resequencing of the evolved strain M3

To identify the genetic basis of the improved carotenoids yield, whole-genome resequencing of strain BL03-D-4 and M3 was performed using the Illumina HiSeq platform through paired-end sequencing. Coverage depth was approximately 70 to 90 reads. Overall, 14 single nucleotide polymorphisms (SNPs) and 100 insertion and deletions (INDEL) were detected in M3, compared to BL03-D-4 after the evolution process (full mutation lists are available in Additional file 2). The resequencing results suggested that M3 had acquired a loss-of-function mutation of *PFK1* since M3 generated a stop codon in the front part of the *PFK1* gene, which encoded 6-phosphofructokinase 1. It has been reported that PFK1 was involved in glycolysis and gluconeogenesis which was related to NADPH generation (Kwak et al. 2020). Furthermore, the *E. coli* central metabolic network was rewired after deletion of *pfkA* which indicated that reduced glycolysis by weakening 6-phosphofructokinase had a profound effect on metabolism (Hollinshead et al. 2016). Thus, combined with the genome resequencing results, we speculated that the loss-of-function mutation of *PFK1* in M3 was related to the promoted carotenoid yield.

### Determining causal mutation

**Fig. 3:**
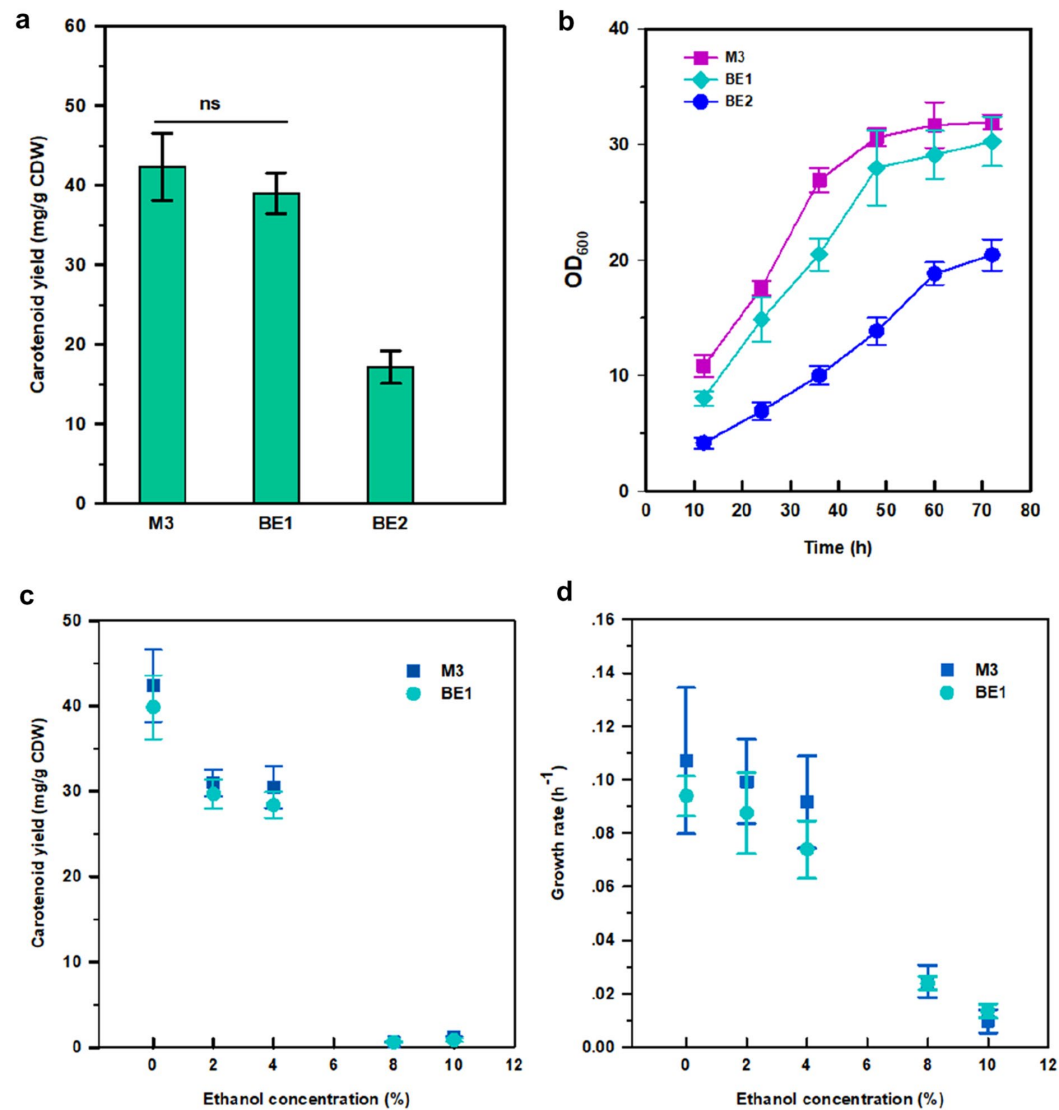
Carotenoid yields (**a**) of evolved (M3), and knockout strains (BE1 and BE2), and growth patterns (**b**) of M3, BE1, and BE2. Effect of different concentrations of ethanol on carotenoid yields of M3 and BE1 (**c**). Specific growth rates of M3 and BE1 under different concentrations of ethanol (**d**). These strains were, respectively, grown in the YPM media at 30 °C for 96 h. Error bars represent standard deviations of three replicates. Two-tailed Student’s *t* test was carried out, ns (not significant)

To determine whether the loss-of-function mutation of *PFK1* confers the improvement of carotenoids yield, the inactivation of *PFK1* was reverse-engineered into BL03-D-4. The reverse-engineered strain BE1 showed remarkably increased carotenoid yield relative to BL03-D-4, which was not significantly different compared to M3 (Fig. 3) without ethanol or under different concentrations of ethanol, fully recovering the evolved carotenoids phenotype (Additional file 1: Figure S3). BE1 represented a similar colonial morphology compared with M3, but with a different morphology to parental strain BL03-D-4 (Additional file 1: Figure S1 and S4). Deletion of *PFK1* had little effect on the growth of strain BE1 without ethanol or under different concentration of ethanol (Fig. 3), these results suggested that mutations other than *PFK1* mutation contributed to the normal growth of M3 and counteracted the negative effect of the loss-of-function mutation of *PFK1*. Since there was an isoenzyme (PFK2) of PFK1 in *S. cerevisiae*, BL03-D-4 was engineered with the deletion of *PFK2* to generate BE2 to test whether *PFK2* mutation has any similar effect on the pentose phosphate pathway (PPP). As shown in Fig. 3b, *PFK2* deletion exerted a severe suppression on the growth of BE2 and no increase of carotenoids was detected in this strain after 96 h fermentation. However, BE2 represented a similar colonial morphology compared with M3 and BE1 after long cultivation (about 8 days) (Additional file 1: Figure S4). These results suggested that PFK2 might be the key enzyme responsible for the conversion of glucose-6-phosphate (G6D) to fructose 1,6-bisphosphatase (FBP) in *S. cerevisiae* which was different compared to *E. coli* (Hollinshead et al. 2016). Morphology observation of BL03-D-4, M3, BE1, and BE2 was carried out through a microscope at 1000 × magnification. These pictures showed that evolved (M3) and knockout strains (BE1 and BE2) presented a larger size of morphology compared to the parental strain (BL03-D-4) (Additional file 1: Figure S5). This change of morphology might be responsible for the promoted carotenoid accumulation.

### Transcriptome analysis of evolved strain M3

**Fig. 4:**
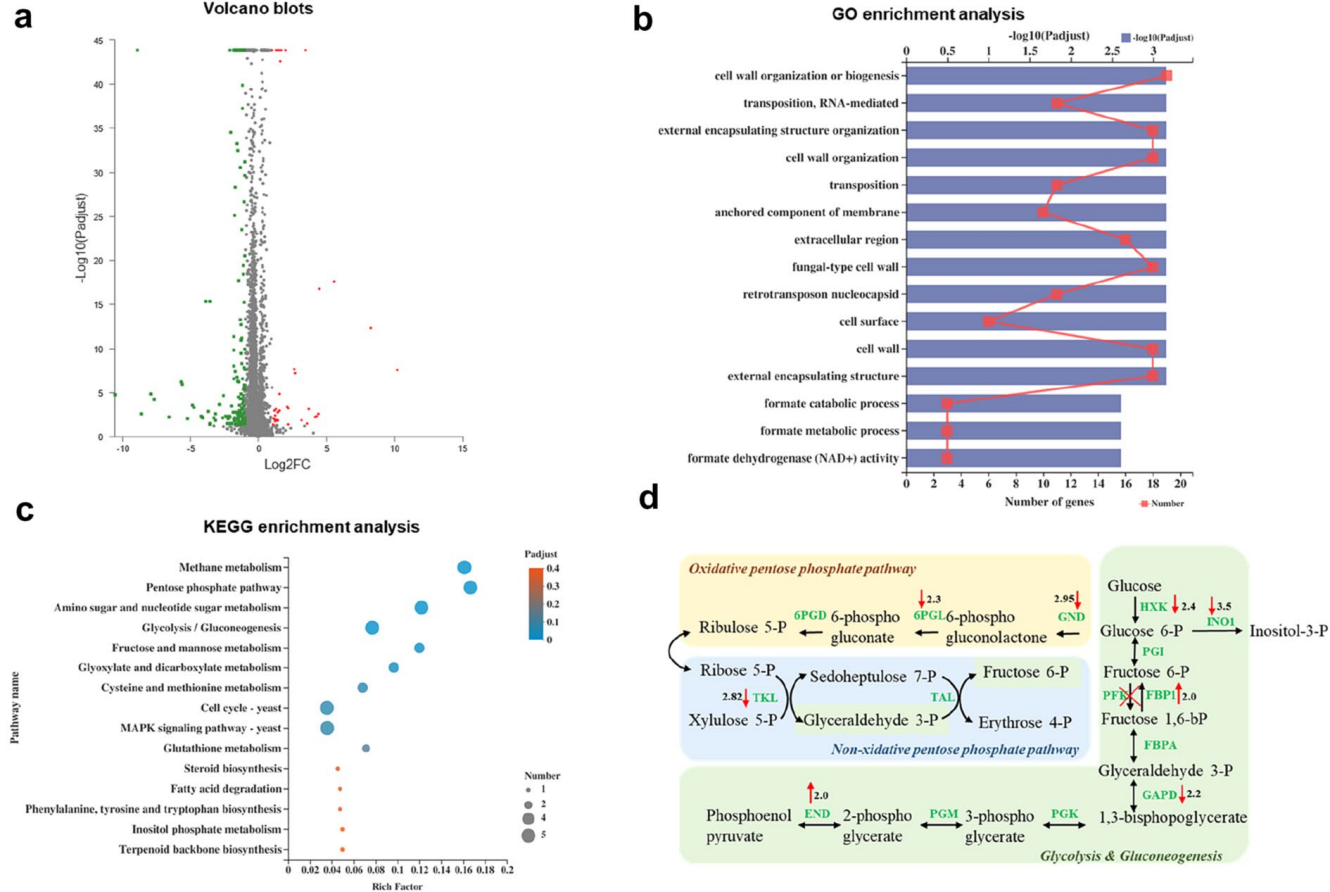
Differentially expressed genes generated by transcriptome analysis in YPM media after 24 h of incubation. **a** Volcano plot of the differentially expressed genes in M3. The horizontal coordinate represented the fold change, and the vertical coordinate represented a significant difference Padjust (logarithmic transformation at the base of 10). Red dots represented up-regulated genes, and green dots represented down-regulated genes. **b**, **c** Histogram of GO enrichment and KEGG pathway enrichment analysis. The vertical coordinate shows the enriched GO terms and the pathway names, and the horizontal coordinate represents the number of genes and Padjust of differentially expressed genes in the term and the pathway, respectively. **d** Overview of significantly changed genes in metabolic pathways. Pentose phosphate pathway (oxidative phase, yellow; non-oxidative phase, blue): GND, glucose 6-phosphate dehydrogenase; 6PGL, 6-phosphogluconolactonase; 6PGD, 6-phosphogluconate dehydrogenase; TKL, transketolase; TAL, transaldolase. Glycolysis and gluconeogenesis (green): HXK, hexokinase; PGI, phosphoglucose isomerase; INO1, inositol-3-phosphate synthase; PFK, phosphofructokinase; FBP1, fructose 1,6-bisphosphatase; FBPA, fructose-bisphosphate aldolase; GAPD: glyceraldehyde phosphate dehydrogenase; PGK, phosphoglycerate kinase; PGM, phosphoglycerate mutase; ENO, enolase

To gain insights into the phenotypic changes that were generated during the ALE process to improved carotenoids yield, we performed a transcriptome analysis of BL03-D-4 and M3. For the transcriptome analysis, strains were cultured in YPM media without the addition of ethanol, and cells were collected after 24 h. We defined the gene sets that showed greater than twofold differences in expression levels between BL03-D-4 and M3. There were 37 up-regulated genes and 162 down-regulated genes in M3 compared with BL03-D-4 through differential expression analysis (Fig. 4a, full lists are available in Additional file 3). We also screened gene functions using GO classification and KEGG enrichment and found enrichment in both up-regulated and down-regulated gene sets in M3 (Fig. 4). KEGG pathway enrichment analysis was executed to further explore the cause of *PFK1* mutation promoting carotenoids accumulation (Fig. 4c). The differentially expressed genes were primarily enriched in the glycolysis/gluconeogenesis pathway and the majority were down-regulated indicating that the loss-of-function mutation of *PFK1* might change G6P biosynthesis in *S. cerevisiae* and regulate the cell wall systems involved in carotenoid accumulation. Furthermore, the GO enrichment analysis demonstrated that the differentially expressed genes were mainly involved in cell wall organization, and almost all genes were down-regulated in this study (Fig. 4b). These results indicated that there might be a relationship between *PFK1* mutation-improved carotenoid accumulation and the cell wall systems.

## Discussion

Currently, there are hundreds of genes involved in ethanol stress, including glycolysis, ethanol metabolism, plasma membrane composition and cell wall biogenesis in *S. cerevisiae* (Ma and Liu 2010). Ethanol resistance was a complex phenotype regulated by multiple genes, in addition to the molecular genetics for enhancing *S. cerevisiae* ethanol tolerance such as global transcription machinery engineering (Alper et al. 2006), transposon mutation (Kim et al. 2011), genome shuffling (Snoek et al. 2015), and metabolic engineering (Lam et al. 2014). The ALE was also used as a conventional approach to improve the ethanol tolerance (Voordeckers et al. 2015). Ma et al. also found that many genes relating to cell wall composition were vital for cell wall organization and most of them were down-regulated under ethanol pressure. They proposed that cell wall structures might undergo significant remodeling processes in response to ethanol stress (Ma and Liu 2010). Furthermore, a crucial factor for the ratio of glucan and mannan in the walls could be the direction of the glucose 6-phosphate/mannose 6-phosphate interconversion (Kratky et al. 1975).

As described above, the loss-of-function mutation of *PFK1* was identified in M3. Previous researches showed that PFK1 was involved in glycolysis and gluconeogenesis (Tripodi et al. 2015). In *E. coli*, two isoenzymes (pfkA and pfkB) referred to the phosphofructokinase and pfkA was considered to be the key enzyme accounting for the conversion of G6P to FBP. The deletion of *pfkA* prolonged lag phase, impaired both cell growth and acetate overflow, accumulated G6P, relieved glucose catabolite repression, and alleviated the Embden–Meyerhof pathway (EMP) repression on gluconeogenesis. The glycolytic flux redistribution resulted in metabolic burdens, cofactor imbalances, and decreasing carbon yield (Hollinshead et al. 2016). Similarly, it was reported that the EMP was replaced (deletion of PGI) with the Entner–Doudoroff pathway (EDP) and oxidative PPP to boost isoprenoid biosynthesis, along with overexpression of *zwf* and *pgl* genes, leading to a 104% squalene increase in *E. coli* (Xu et al. 2019). However, there is little reference to *PFK1* mutation in *S. cerevisiae*.

To clarify the effect of the inactivation of PFK1 on cell metabolism, we screened the genes whose expression levels were dramatically changed in M3. As shown in Fig. 4d and Additional file 1: Table S3, M3 generally showed lower expression levels in the PPP and cell wall-related genes than BL03-D-4. NADPH generation was highly reliant on the oxidative PP pathway. The metabolic flux toward the oxidative PPP was always limited due to the rigid glycolysis flux in *S. cerevisiae* (Minard and McAlister-Henn 2005; Zampar et al. 2013). Therefore, the increase of G6P fluxes toward the oxidative PPP through glucose-6-phosphate dehydrogenase (G6PD), instead of glycolysis, was necessary for efficient NADPH production and enhanced production of isoprenoids (Kwak et al. 2020). Similarly, efficient carotenoid biosynthesis needed NADPH providing reducing power (Hong et al. 2019; Zhao et al. 2015). Surprisingly, and in contrast, the first and second steps of the oxidative PPP were significantly down-regulated in M3, which presented a remarkable improvement of carotenoid yield in our study (Fig. 4d). This result suggested that the loss-of-function mutation of *PFK1* might contribute to a new mechanism for improving carotenoid yield. For resisting the ethanol pressure, the variation of membrane fluidity was the major way in *S. cerevisiae* (Wang et al. 2018; Yang et al. 2019). In this study, it might be the cell wall remodeling that mainly stands up to ethanol stress. For cell wall biogenesis, many genes involved in cell wall organization were down-regulated without ethanol pressure in M3 (Additional file 1: Table S3). It is worth mentioning that thickened cell walls and larger yeast were observed in the micafungin resistant yeast (Li et al. 2014). Furthermore, secretory pathways transported cell wall proteins onto the plasma membrane, as well as transferring lipids, via vesicles, to repair membrane destruction under ethanol stress and they might contribute to ethanol tolerance in *Kluyveromyces marxianus* (Mo et al. 2019). Changes of the cell wall might be responsible for the change of membranes, which further affected the storage ability of those fat-soluble carotenoids. The strategy based on this probable mechanism could supplement the previously reported approaches about improving carotenoid yield in *S. cerevisiae.*

## Conclusions

In conclusion, a new ethanol-induced ALE was successfully applied to improve carotenoid yield in engineered *S. cerevisiae* and a hyper-producer was isolated from evolution with a 5.1-fold increase in carotenoid yield. The loss-of-function mutation of *PFK1* was revealed as being the cause of increased carotenoid yield through whole-genome resequencing and reverse engineering. Transcriptomic analysis revealed the strengthening of gluconeogenesis and downregulation of cell wall-related genes, as a potential perturbation for the improvement of carotenoid yield. This study provided a classic case where the appropriate selective pressure could be employed to improve carotenoid yield using ALE and identified a novel metabolic engineering target *PFK1* for carotenoid production in *S. cerevisiae*.

### Supplementary Information

**Additional file 1:** **Table S1.** Strains used in this study. **Table S2.** Primers used in this study. **Table S3.** Differential expression of cell wall‐related genes. **Figure S1.** BL03-D-4 and M3 on YPD plates. **Figure S2.** Shake-flask fermentations of BL03-D-4 and M3 in YPM medium with different concentration of ethanol. **Figure S3.** Shake-flask fermentations of BL03-D-4, M3 and BE1 in YPM medium and YPD medium. **Figure S4.** Colony morphology of BL03-D-4, M3, BE1 and BE2. Figure S5 Morphology observation of BL03-D-4, M3, BE1 and BE2.

**Additional file 2:** Full mutation lists of SNPs and INDEL.

**Additional file 3:** Differential expression analysis.

## Data Availability

Fastq DNA-seq raw data were deposited in the Genome Sequence Archive (GSA) server at the BIG Data Center in Beijing Institute of Genomics (http://bigd.big.ac.cn, GSA accession No. CRA003704), RNA-seq raw data were deposited into NCBI (GEO accession number GSE164470). The dataset generated during and/or analyzed during the current study are available from the corresponding author on reasonable request. Strain M3 was deposited at Guangdong Microbial Culture Collection Center (GDMCC No. 61336). The materials that support the findings of this study are available from the corresponding author on request.

## Abbreviations

ARTP: Atmospheric and room temperature plasma
ALE: Adaptive laboratory evolution
PFK1: Phosphofructokinase 1
YPM: Modified YPD medium
TMS: Trace metal solution
GSA: Genome Sequence Archive
CDW: Cell dry weight
SNPs: Single nucleotide polymorphisms
INDEL: Insertion and deletions
PPP: Pentose phosphate pathway
G6P: Glucose-6-phosphate
FBP: Fructose 1,6-bisphosphatase
EMP: Embden–Meyerhof pathway
EDP: Entner–Doudoroff pathway
G6PD: Glucose-6-phosphate dehydrogenase

## References

[CR1] Alper H, Moxley J, Nevoigt E, Fink GR, Stephanopoulos G (2006). Engineering yeast transcription machinery for improved ethanol tolerance and production. Science.

[CR2] Chen Y, Xiao W, Wang Y, Liu H, Li X, Yuan Y (2016). Lycopene overproduction in *Saccharomyces cerevisiae* through combining pathway engineering with host engineering. Microb Cell Fact.

[CR3] Dragosits M, Mattanovich D (2013). Adaptive laboratory evolution—principles and applications for biotechnology. Microb Cell Fact.

[CR4] Godara A, Rodriguez MAG, Weatherston JD, Peabody GL, Wu HJ, Kao KC (2019). Beneficial mutations for carotenoid production identified from laboratory-evolved *Saccharomyces cerevisiae*. J Ind Microbiol Biotechnol.

[CR5] Guo L, Pang Z, Gao C, Chen X, Liu L (2020). Engineering microbial cell morphology and membrane homeostasis toward industrial applications. Curr Opin Biotechnol.

[CR6] Hollinshead WD, Rodriguez S, Martin HG, Wang G, Baidoo EE, Sale KL, Keasling JD, Mukhopadhyay A, Tang YJ (2016). Examining Escherichia coli glycolytic pathways, catabolite repression, and metabolite channeling using *Delta pfk* mutants. Biotechnol Biofuels.

[CR7] Hong J, Park SH, Kim S, Kim SW, Hahn JS (2019). Efficient production of lycopene in *Saccharomyces cerevisiae* by enzyme engineering and increasing membrane flexibility and NAPDH production. Appl Microbiol Biotechnol.

[CR8] Jiang G, Yang Z, Wang Y, Yao M, Chen Y, Xiao W, Yuan Y (2020). Enhanced astaxanthin production in yeast via combined mutagenesis and evolution. Biochem Eng J.

[CR9] Jin J, Wang Y, Yao M, Gu X, Li B, Liu H, Ding M, Xiao W, Yuan Y (2018). Astaxanthin overproduction in yeast by strain engineering and new gene target uncovering. Biotechnol Biofuels.

[CR10] Kim HS, Kim NR, Yang J, Choi W (2011). Identification of novel genes responsible for ethanol and/or thermotolerance by transposon mutagenesis in *Saccharomyces cerevisiae*. Appl Microbiol Biotechnol.

[CR11] Kratky Z, Biely P, Bauer S (1975). Mechanism of 2-deoxy-D-glucose inhibition of cell-wall polysaccharide and glycoprotein biosyntheses in *Saccharomyces cerevisiae*. Eur J Biochem.

[CR12] Kwak S, Yun EJ, Lane S, Oh EJ, Kim KH, Jin YS (2020). Redirection of the glycolytic flux enhances isoprenoid production in *Saccharomyces cerevisiae*. Biotechnol J.

[CR13] Lam FH, Ghaderi A, Fink GR, Stephanopoulos G (2014). Biofuels. Engineering alcohol tolerance in yeast. Science.

[CR14] Lee S, Kim P (2020). Current status and applications of adaptive laboratory evolution in industrial microorganisms. J Microbiol Biotechnol.

[CR15] Li X-E, Wang J-J, Phornsanthia S, Yin X, Li Q (2014). Strengthening of cell wall structure enhances stress resistance and fermentation performance in lager yeast. J Am Soc Brew Chem.

[CR16] Ma M, Liu ZL (2010). Mechanisms of ethanol tolerance in *Saccharomyces cerevisiae*. Appl Microbiol Biotechnol.

[CR17] Ma T, Shi B, Ye Z, Li X, Liu M, Chen Y, Xia J, Nielsen J, Deng Z, Liu T (2019). Lipid engineering combined with systematic metabolic engineering of *Saccharomyces cerevisiae* for high-yield production of lycopene. Metab Eng.

[CR18] Minard KI, McAlister-Henn L (2005). Sources of NADPH in yeast vary with carbon source. J Biol Chem.

[CR19] Mo W, Wang M, Zhan R, Yu Y, He Y, Lu H (2019). *Kluyveromyces marxianus* developing ethanol tolerance during adaptive evolution with significant improvements of multiple pathways. Biotechnol Biofuels.

[CR20] Mussagy CU, Winterburn J, Santos-Ebinuma VC, Pereira JFB (2019). Production and extraction of carotenoids produced by microorganisms. Appl Microbiol Biotechnol.

[CR21] Reyes LH, Gomez JM, Kao KC (2014). Improving carotenoids production in yeast via adaptive laboratory evolution. Metab Eng.

[CR22] Saini RK, Keum YS (2019). Microbial platforms to produce commercially vital carotenoids at industrial scale: an updated review of critical issues. J Ind Microbiol Biotechnol.

[CR23] Sandberg TE, Salazar MJ, Weng LL, Palsson BO, Feist AM (2019). The emergence of adaptive laboratory evolution as an efficient tool for biological discovery and industrial biotechnology. Metab Eng.

[CR24] Shi B, Ma T, Ye Z, Li X, Huang Y, Zhou Z, Ding Y, Deng Z, Liu T (2019). Systematic metabolic engineering of *Saccharomyces cerevisiae* for lycopene overproduction. J Agric Food Chem.

[CR25] Snoek T, Picca Nicolino M, Van den Bremt S, Mertens S, Saels V, Verplaetse A, Steensels J, Verstrepen KJ (2015). Large-scale robot-assisted genome shuffling yields industrial *Saccharomyces cerevisiae* yeasts with increased ethanol tolerance. Biotechnol Biofuels.

[CR26] Su B, Li A, Deng MR, Zhu H (2021). Transcriptome analysis reveals a promotion of carotenoid production by copper ions in recombinant *Saccharomyces cerevisiae*. Microorganisms.

[CR27] Su B, Song D, Yang F, Zhu H (2020). Engineering a growth-phase-dependent biosynthetic pathway for carotenoid production in *Saccharomyces cerevisiae*. J Ind Microbiol Biotechnol.

[CR28] Su B, Song D, Zhu H (2020). Metabolic engineering of *Saccharomyces cerevisiae* for enhanced carotenoid production from xylose-glucose mixtures. Front Bioeng Biotechnol.

[CR29] Tripodi F, Nicastro R, Reghellin V, Coccetti PR (2015). Post-translational modifications on yeast carbon metabolism: regulatory mechanisms beyond transcriptional control. Biochim Biophys Acta.

[CR30] Voordeckers K, Kominek J, Das A, Espinosa-Cantu A, De Maeyer D, Arslan A, Van Pee M, van der Zande E, Meert W, Yang Y, Zhu B, Marchal K, DeLuna A, Van Noort V, Jelier R, Verstrepen KJ (2015). Adaptation to high ethanol reveals complex evolutionary pathways. PLoS Genet.

[CR31] Wang S, Sun X, Yuan Q (2018). Strategies for enhancing microbial tolerance to inhibitors for biofuel production: a review. Bioresour Technol.

[CR32] Wang C, Zhao S, Shao X, Park JB, Jeong SH, Park HJ, Kwak WJ, Wei G, Kim SW (2019). Challenges and tackles in metabolic engineering for microbial production of carotenoids. Microb Cell Fact.

[CR33] Xie W, Ye L, Lv X, Xu H, Yu H (2015). Sequential control of biosynthetic pathways for balanced utilization of metabolic intermediates in *Saccharomyces cerevisiae*. Metab Eng.

[CR34] Xu W, Yao J, Liu L, Ma X, Li W, Sun X, Wang Y (2019). Improving squalene production by enhancing the NADPH/NADP(+) ratio, modifying the isoprenoid-feeding module and blocking the menaquinone pathway in *Escherichia coli*. Biotechnol Biofuels.

[CR35] Yang Y, Xia Y, Hu W, Tao L, Ni L, Yu J, Ai L (2019). Membrane Fluidity of *Saccharomyces cerevisiae* from Huangjiu (Chinese Rice Wine) Is Variably Regulated by *OLE1* To Offset the Disruptive Effect of Ethanol. Appl Environ Microbiol.

[CR36] Zampar GG, Kummel A, Ewald J, Jol S, Niebel B, Picotti P, Aebersold R, Sauer U, Zamboni N, Heinemann M (2013). Temporal system-level organization of the switch from glycolytic to gluconeogenic operation in yeast. Mol Syst Biol.

[CR37] Zhao X, Shi F, Zhan W (2015). Overexpression of *ZWF1* and *POS5* improves carotenoid biosynthesis in recombinant *Saccharomyces cerevisiae*. Lett Appl Microbiol.

[CR38] Zhu Z, Zhang J, Ji X, Fang Z, Wu Z, Chen J, Du G (2018). Evolutionary engineering of industrial microorganisms-strategies and applications. Appl Microbiol Biotechnol.

