# Supplementary material for: Identification of a novel metabolic engineering target for carotenoid production in Saccharomyces cerevisiae via ethanol-induced adaptive laboratory evolution
Source: Bioresour Bioprocess. 2021 Jun 11;8(1):47. doi: 10.1186/s40643-021-00402-5 (PMC10992865; doi:10.1186/s40643-021-00402-5)
Supplement: Supplementary file 1 — Additional file 1: Table S1. Strains used in this study. Table S2. Primers used in this study. Table S3. Differential expression of cell wall‐related genes. Figure S1. BL03-D-4 and M3 on YPD plates. Figure S2. Shake-flask fermentations of BL03-D-4 and M3 in YPM medium with different concentration of ethanol. Figure S3. Shake-flask fermentations of BL03-D-4, M3 and BE1 in YPM medium and YPD medium. Figure S4. Colony morphology of BL03-D-4, M3, BE1 and BE2. Figure S5 Morphology observation of BL03-D-4, M3, BE1 and BE2. [file 40643_2021_402_MOESM1_ESM.docx]

**Supplementary Information**

**Additional file 1**

**Identification of a novel metabolic engineering target for** **carotenoid** **production in *Saccharomyces cerevisiae*** **via** **ethanol-induced** **adaptive laboratory evolution**

Buli Su, Anzhang Li, Ming-Rong Deng*, Honghui Zhu*

Guangdong Microbial Culture Collection Center (GDMCC), Guangdong Provincial Key Laboratory of Microbial Culture Collection and Application, State Key Laboratory of Applied Microbiology Southern China, Institute of Microbiology, Guangdong Academy of Sciences, Guangzhou 510070, People’s Republic of China.

*Corresponding author

Ming-Rong Deng: E-mail: [dengmr@gdim.cn](mailto:dengmr@gdim.cn)

Honghui Zhu: E-mail: [zhuhh@gdim.cn](mailto:zhuhh@gdim.cn)

Tel: +86-020-87137669, Fax: +86-020-87685699

**Table S1** Strains used in this study.

| Strain | Description | Source |
| --- | --- | --- |
| BL03-D-4 | BY4742, Δ*Gal80⸬* *P_HSP26_-CrtB-T_ADH1_-P_HSP26_-CrtI-T_GPM1_-P_HSP26_-CrtE-T_CYC1，_Δ416d⸬* *P_Cit1_-tHMGR-T_Guo,_ ΔAld6* | Su et al. 2020b |
| M3  BE1  BE2 | Evolved strain from BL03-D-4  BL03-D-4, *ΔPFK1*  BL03-D-4, *ΔPFK2* | This study  This study  This study |

**Table S2.** Primers used in this study. Homologous overhang-nucleotides (underlined).

| Primers | Sequence (5’-3’) |  |
| --- | --- | --- |
| PFK1-F-2  PFK1-F  PFK1-R  PFK1-R-2  PFK1-CHECK-R  PFK2-F-2  PFK2-F  PFK2-R  PFK2-R-2  PFK2-CHECK-R | Primers for constructing strain BE1 with the deletion of *PFK1*  gaaacaaaatcatatcaaagatgcaatctcaagattcatgctacggtgtt tctcaagattcatgctacggtgttctattactcttggcctcctctagtaca  aactttaatctaccggacaggatgacggaataccacttgccacctatcacc cggctaaagcggctacctcagctctcaactttaatctaccggacaggatg  tgcatgccatttttacctccttttgc  Primers for constructing strain BE2 with the deletion of *PFK2*  agcctttcttatacctcatttgaacaatagaactagatttagagactagt aatagaactagatttagagactagtctattactcttggcctcctctagtaca  atggtcagcaatgagtctggtagacggaataccacttgccacctatcacc  ttaatcaactctctttcttccaaccaaatggtcagcaatgagtctggtag aatattggtttcatggggtagtacttg |  |

**Table S3** Differential expression of cell wall‐related genes.

| Genes | Protein | Description | Fold changes |
| --- | --- | --- | --- |
| *CIS3* | Mannose-containing glycoprotein | Constituent of the cell wall | 0.23 |
| *SRL1*  *GAS1*  *SWC10*  *NCW2 TOS1*  *SCW4*  *EGT2* | Mannoprotein  Beta-1,3-glucanosyltransferase  Cell wall protein  Fungal-type cell wall organization  Covalently-bound cell wall protein  Cell wall protein  Cell wall endoglucanase | Required for cell wall stability in the absence of GPI-anchored mannoproteins  Required for cell wall assembly and also has a role in transcriptional silencing  Plays a role in conjugation during mating  Structural constituent of the cell wall  Cell cycle regulated SBF target gene  Paralog of SWC10  Required for proper cell separation after cytokinesis | 0.24  0.28  0.30  0.32  0.32  0.32  0.43 |
| *PUN1*  *CCW12*  *PSA1*  *SUN4*  *TIP1* | Plasma membrane protein  Cell wall mannoprotein  Mannose-1-phosphate guanyltransferase  Cell wall protein  Major cell wall mannoprotein | Plays a role in cell wall integrity  Plays a role in maintenance of newly synthesized areas of cell wall  Required for normal cell wall structure    Involved in cell wall septation  Lipase activity | 0.44  0.45  0.46  0.48  2.53 |


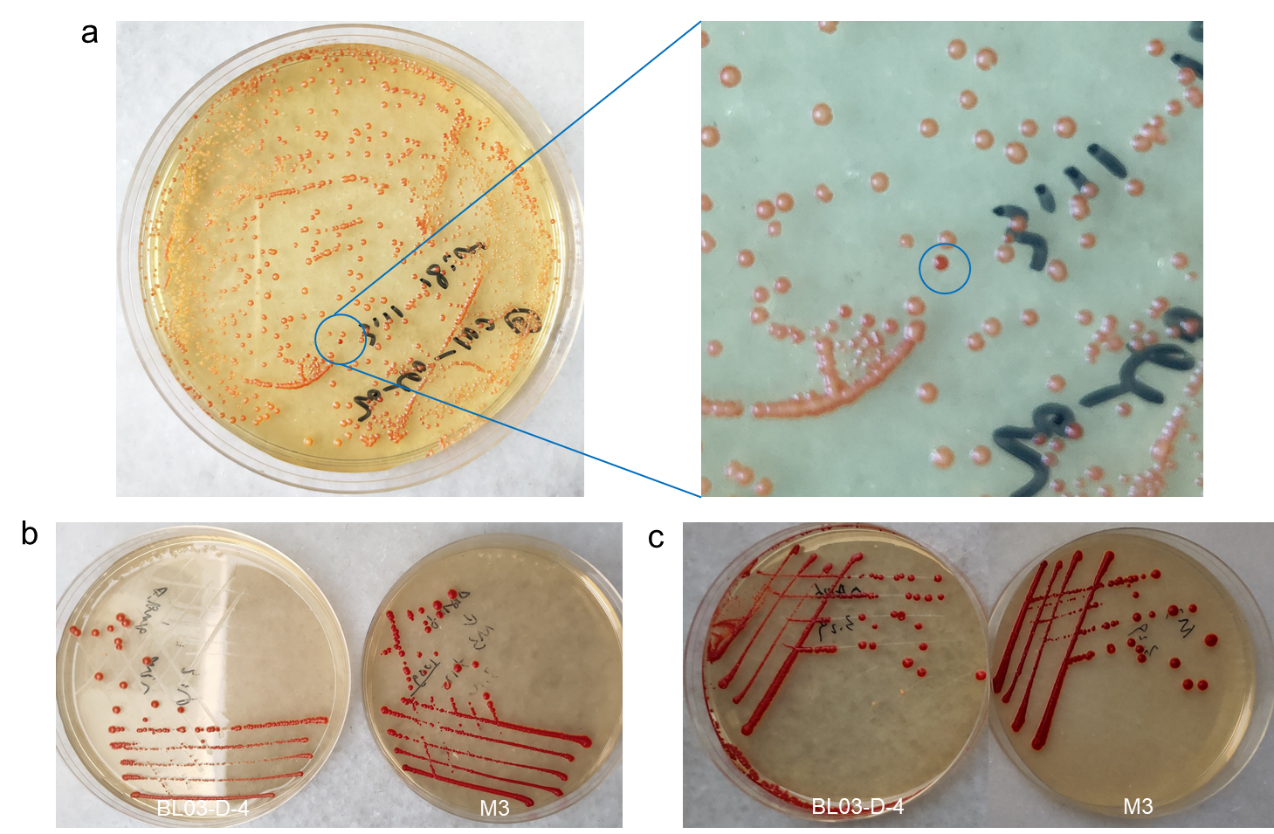


**Figure S1** Ethanol-induced adaptive laboratory evolution was successfully applied for improving carotenoids yield in engineered *S. cerevisiae* and hyper-producer M3 was isolated (a). BL03-D-4 and M3 were cultured on YPD plates for 48 (b) or 96 h (c).


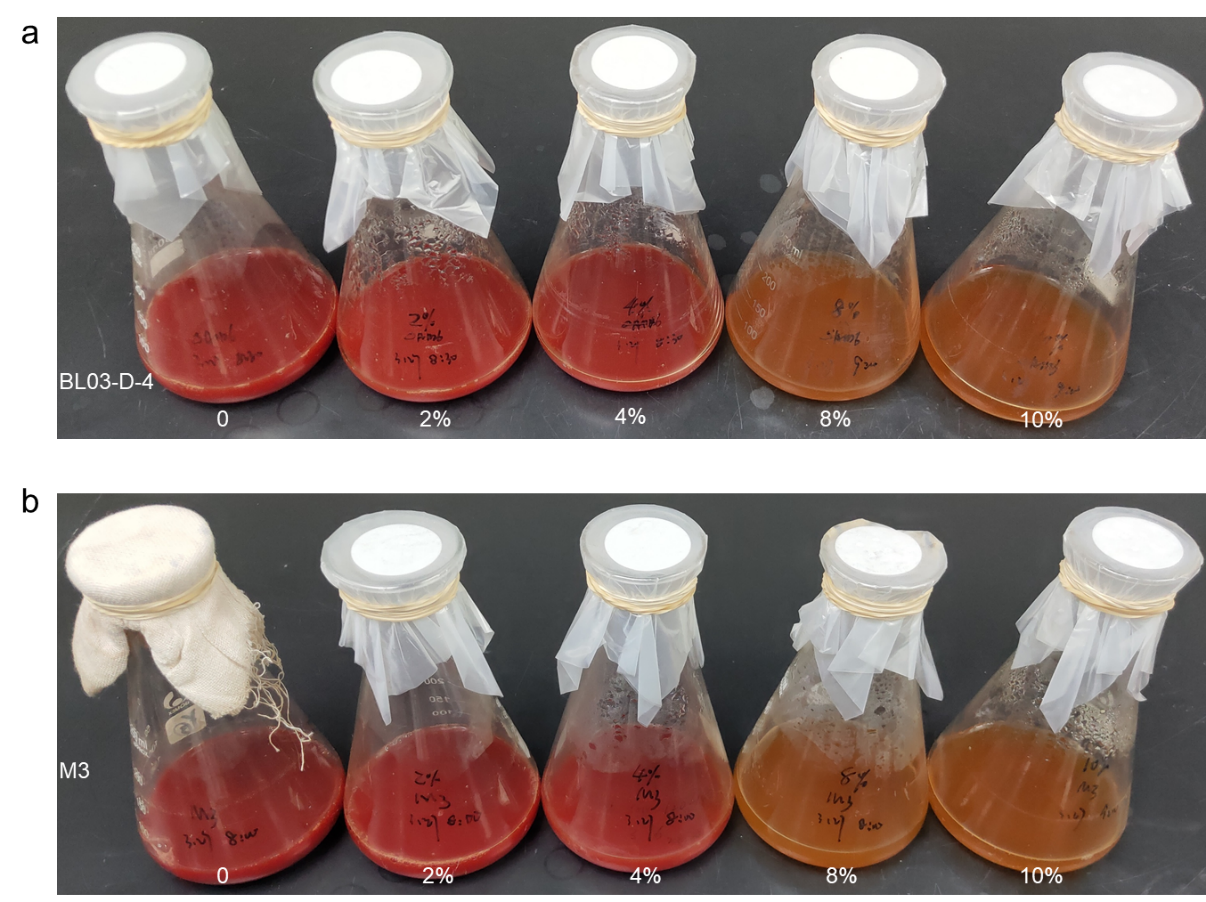


**Figure S2** Shake-flask fermentations of BL03-D-4 (a) and M3 (b) in YPM medium with different concentration of ethanol for 96 h.


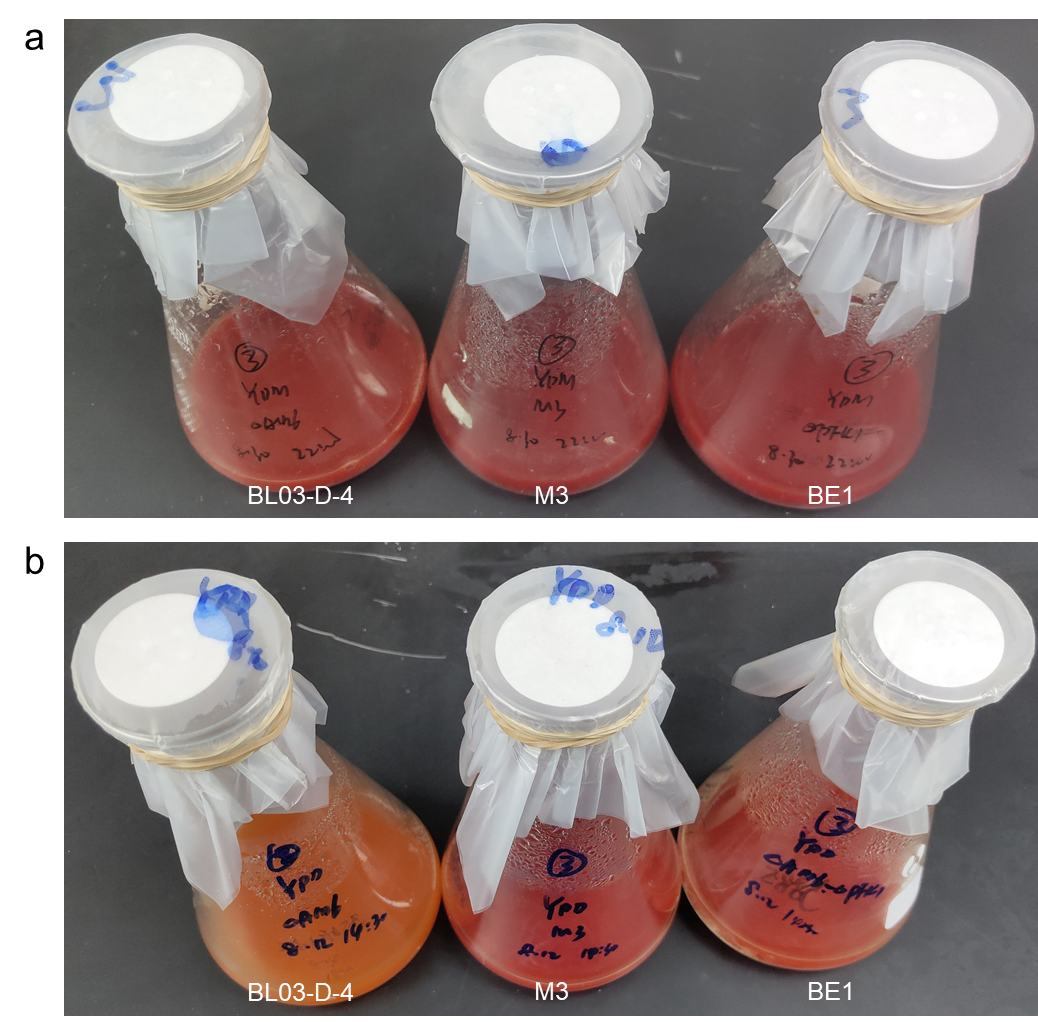


**Figure S3** Shake-flask fermentations of BL03-D-4, M3 and BE1 in YPM medium (a) and YPD medium (b) for 96 h.


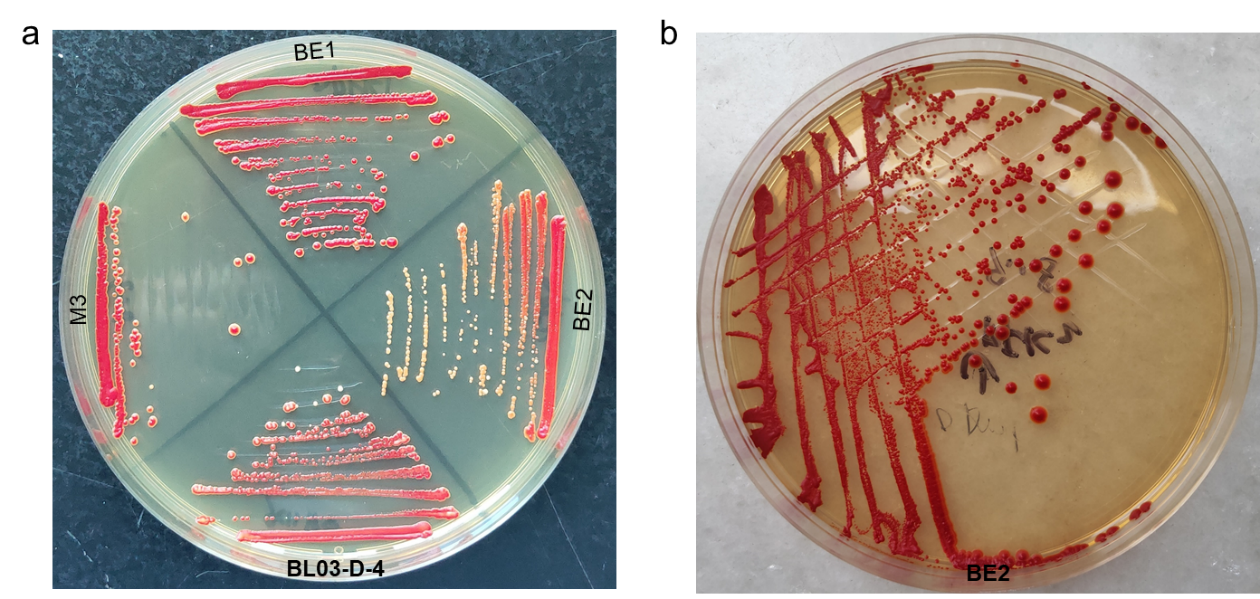


**Figure S4** Colony morphology of BL03-D-4, M3, BE1 and BE2. These strains were cultured on YPD plate for 48 h (a). BE2 was cultured on YPD plate for 8 days (b).


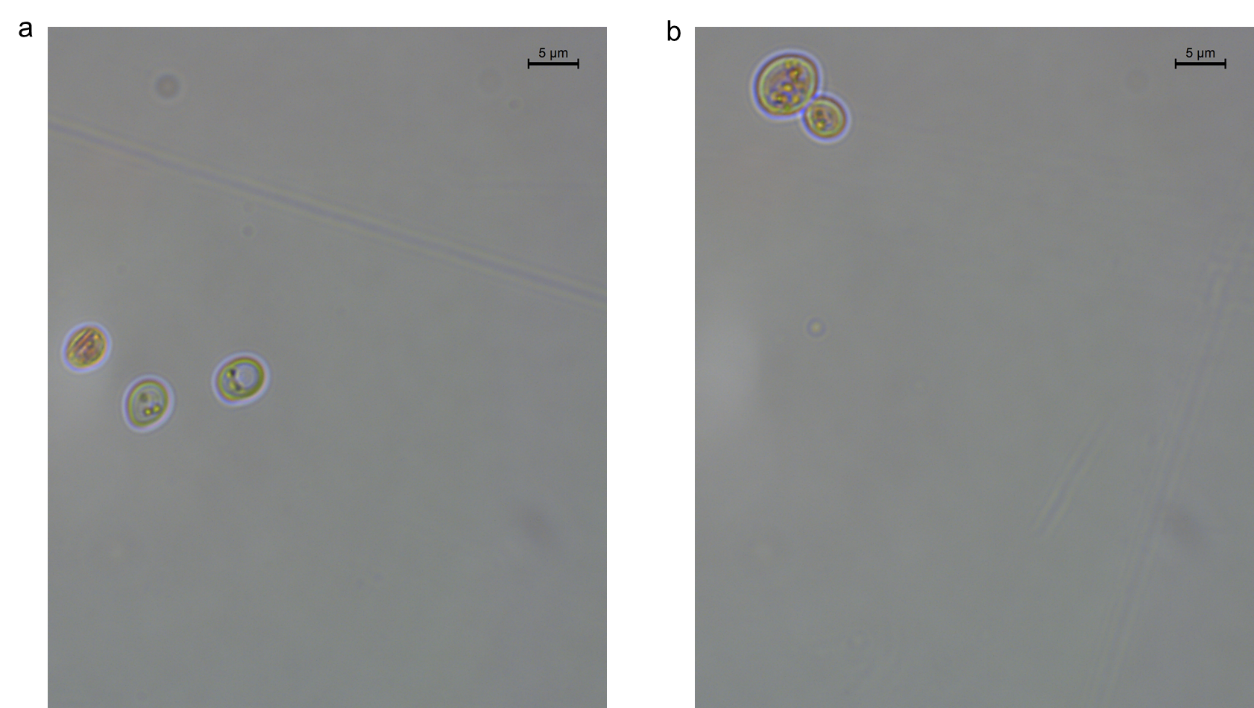


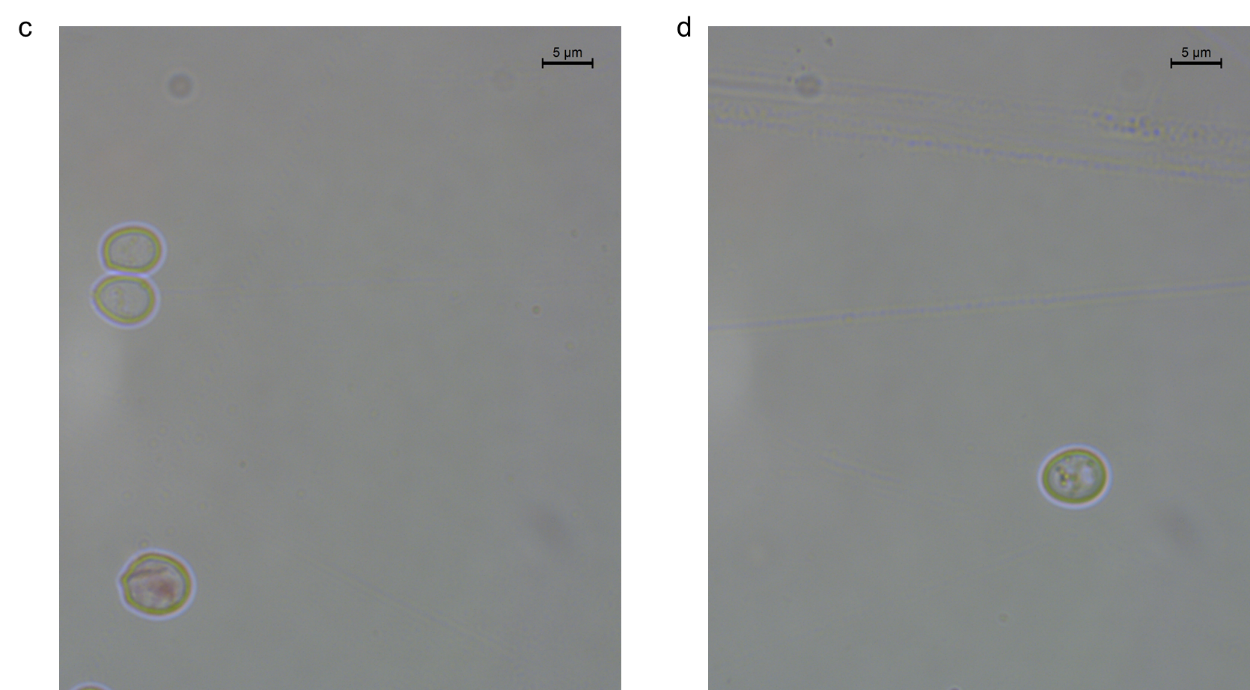

**Figure S5** Morphology observation of BL03-D-4 (a), M3 (b), BE1 (c) and BE2 (d) were carried out by using microscope at 1000× magnification. The major axis of different strains. ***: significantly different based on two-tailed Student’s t-test (*p* < 0.001), ns: not significant. These strains were respectively grown on the YPD plates at 30 °C for 48 h.
